# Leloir glycosyltransferases enabled to flow synthesis: Continuous production of the natural *C*‐glycoside nothofagin

**DOI:** 10.1002/bit.27908

**Published:** 2021-08-16

**Authors:** Hui Liu, Bernd Nidetzky

**Affiliations:** ^1^ Institute of Biotechnology and Biochemical Engineering Graz University of Technology, NAWI Graz Graz Austria; ^2^ Austrian Centre of Industrial Biotechnology (ACIB) Graz Austria

**Keywords:** continuous production, enzyme co‐immobilization, flow bio‐catalysis, glycosylation, natural product glycosides, process intensification, sugar nucleotide‐dependent glycosyltransferase

## Abstract

*C*‐glycosyltransferase (CGT) and sucrose synthase (SuSy), each fused to the cationic binding module *Z*_basic2_, were co‐immobilized on anionic carrier (ReliSorb SP400) and assessed for continuous production of the natural *C*‐glycoside nothofagin. The overall reaction was 3ʹ‐*C*‐*β*‐glycosylation of the polyphenol phloretin from uridine 5ʹ‐diphosphate (UDP)‐glucose that was released in situ from sucrose and UDP. Using solid catalyst optimized for total (∼28 mg/g) as well as relative protein loading (CGT/SuSy = ∼1) and assembled into a packed bed (1 ml), we demonstrate flow synthesis of nothofagin (up to 52 mg/ml; 120 mM) from phloretin (≥95% conversion) solubilized by inclusion complexation in hydroxypropyl *β*‐cyclodextrin. About 1.8 g nothofagin (90 ml; 12–26 mg/ml) were produced continuously over 90 reactor cycles (2.3 h/cycle) with a space‐time yield of approximately 11 mg/(ml h) and a total enzyme turnover number of up to 2.9 × 10^3^ mg/mg (=3.8 × 10^5^ mol/mol). The co‐immobilized enzymes exhibited useful effectiveness (∼40% of the enzymes in solution), with limitations on the conversion rate arising partly from external liquid–solid mass transfer of UDP under packed‐bed flow conditions. The operational half‐life of the catalyst (∼200 h; 30°C) was governed by the binding stability of the glycosyltransferases (≤35% loss of activity) on the solid carrier. Collectively, the current study shows integrated process technology for flow synthesis with co‐immobilized sugar nucleotide‐dependent glycosyltransferases, using efficient glycosylation from sucrose via the internally recycled UDP‐glucose. This provides a basis from engineering science to promote glycosyltransferase applications for natural product glycosides and oligosaccharides.

## Graphical abstract

Sugar nucleotide‐dependent (Leloir) glycosyltransferases are important enzymes of glycoside synthesis, but their development for flow chemistry applications based on heterogeneous bio‐catalysis is lacking. Here, the authors show continuous production of the natural C‐glycoside nothofagin using co‐immobilized C‐glycosyltransferase (Z‐OsCGT) and sucrose synthase (Z‐GmSuSy) in a packed‐bed flow reactor. Cascade reaction to glycosylate phloretin from glucose via intermediary UDP‐glucose is demonstrated at excellent yield, product concentration, space time yield (STY) and total turnover number of enzyme (TTN).

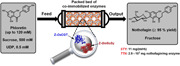

**Abbreviations**
*Gm*SuSy: sucrose synthase from soybean (*Glycine max*)
*Os*CGT: *C*‐glycosyltransferase from rice (*Oryza sativa*)

## 1 INTRODUCTION

Technologies of advanced bioprocessing are regarded as key components of the transition to a sustainable bioeconomy (Aguilar et al., 2019; Sheldon & Woodley, 2018). They involve process intensification as a common target of central importance (Kim et al., 2017; Stankiewicz & Moulijn, 2000; van der Wielen et al., 2021; Woodley, 2017). High‐performance bioproduction systems and continuous processing are main pillars of integrated strategies towards bioprocess intensification (Buchholz et al., 2005; Woodley, 2020; Wu et al., 2021). In biocatalysis applied to chemical synthesis, competitive process technologies build on highly active enzyme preparations that are incorporated efficiently into scalable bioreactors for continuous operation (Buchholz et al., 2005; Cardoso Marques et al., 2021; De Santis et al., 2020; Liese & Hilterhaus, 2013). Among the options available, immobilizing the soluble enzyme(s) on a solid carrier remains in the center of attention for development (Garcia‐Galan et al., 2011; Guisan et al., 2020; Rodrigues et al., 2013). There are excellent opportunities for an integrated design of the catalyst and the reaction (Buchholz et al., 2005; Liese & Hilterhaus, 2013). This is promising in particular with multienzyme cascade reactions that have drawn much interest recently (France et al., 2017; Krasnova & Wong, 2019; W. Q. Li et al., 2019; Riva & Fessner, 2014; Schmid‐Dannert & Lopez‐Gallego, 2019; Schrittwieser et al., 2018). The idea underlying the “cascading” is to gain synthetic efficiency by telescoping multiple enzymatic reactions into a one‐pot overall transformation without the need for intermediary product isolation (Fessner, 2015; Sheldon & Woodley, 2018). While attractive as a concept, its realization for chemical production is challenging, especially under fulfillment of the demand of efficient and robust process technology for continuous operation (Arana‐Peña et al., 2020; Fernandes & de Carvalho, 2021). Enzyme co‐immobilization on porous particles such that individual enzymes are localized in close proximity to each other offers suitable balance between efficiency, flexibility and spatiotemporal control (Bolivar et al., 2017, 2019; Bolivar, Schelch, et al., 2016; Quin et al., 2017; G. Q. Zhang et al., 2018; Zhong et al., 2020). Common tasks in a cascade transformation (e.g., the recycling of co‐substrates and coenzymes; the mass flow from one reaction step to the next) are thus realized efficiently (Caparco et al., 2020; Velasco‐Lozano et al., 2017; Xu et al., 2020). Importantly, besides the essential features of the immobilized enzyme system (e.g., mode of protein tethering; overall protein loading; enzyme activity ratio for flux control) (Bolivar & Nidetzky, 2019, 2020; Hanefeld et al., 2009; Rocha‐Martín et al., 2021; Zhong et al., 2020), the key characteristics of the solid carrier (e.g., surface and bulk material chemistry; external and internal hydrodynamic properties; and mechanical properties) can be tailored to the requirement of the continuous process (Bayne et al., 2013; Liese et al., 2013; Buchholz et al., 2005).

**Scheme 1:**
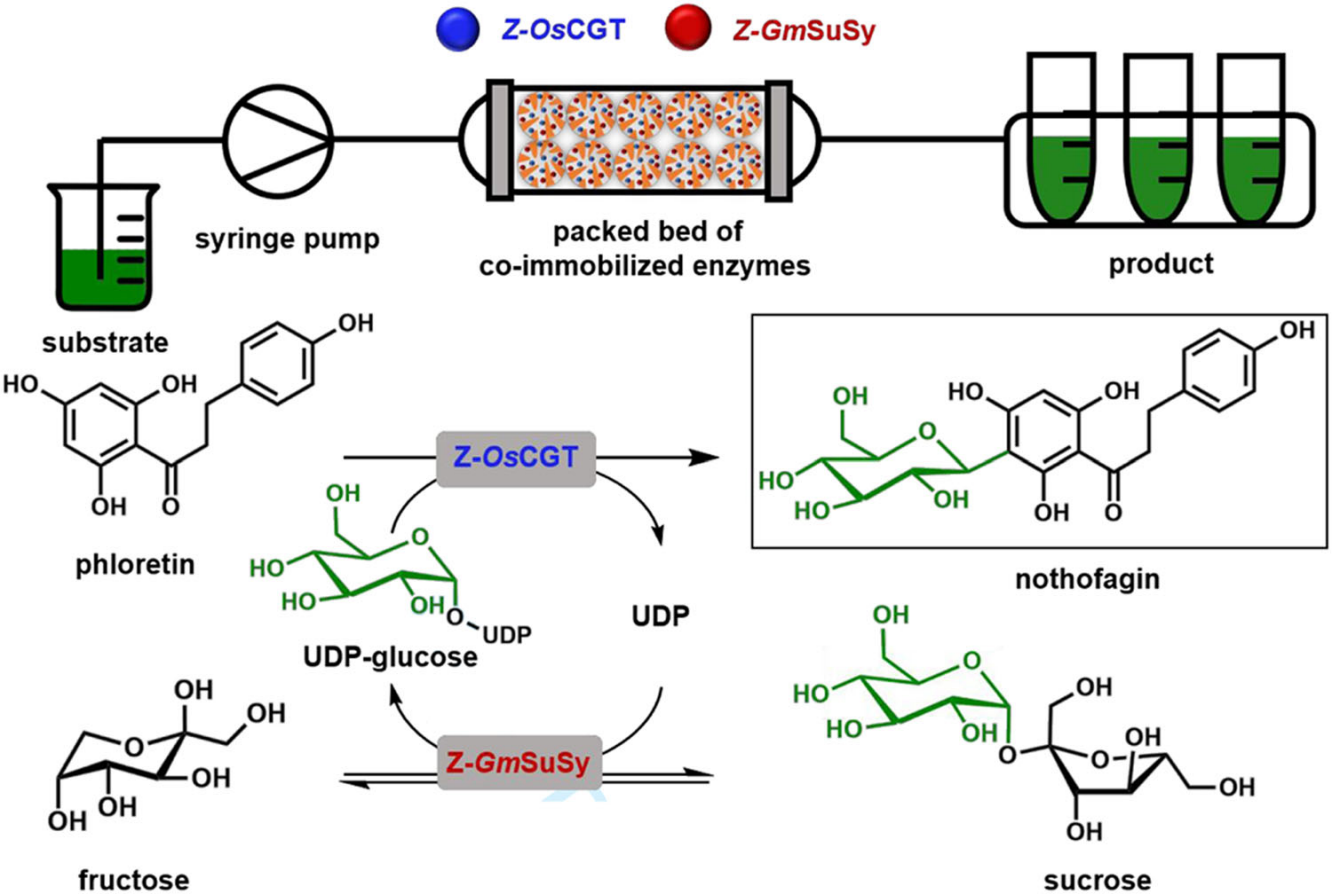
Glycosyltransferase cascade reaction for synthesis of nothofagin and its integration into a packed‐bed reactor format for continuous production

Despite the growing awareness of “flow processing” in applied bio‐catalysis (Britton et al., 2018; De Santis et al., 2020; Tamborini et al., 2018; Thompson et al., 2018; Žnidaršič‐Plazl, 2019), the idea of process intensification via continuous reaction engineering has largely remained foreign to the class of sugar nucleotide‐dependent (Leloir) glycosyltransferases (Nidetzky et al., 2018). These enzymes are powerful catalysts of glycosylation and have considerable importance in the synthesis of natural product glycosides and bioactive oligosaccharides (Mestrom et al., 2019; Na et al., 2021). Semi‐automated flow syntheses of oligosaccharides under use of glycosyltransferases have been reported (Li et al., 2019; Wen et al., 2018; Zhang et al., 2018). However, lacking the essential design from process engineering, the used procedures are not scalable and thus hardly suitable for production. The requirement for a sugar nucleotide substrate, which it is inexpedient to use as a reagent, necessitates that glycosyltransferase reactions are performed as part of a multienzyme cascade transformation (Nidetzky et al., 2018). The important role of the cascade is to establish a catalytic cycle of in situ sugar nucleotide supply. The glycosyltransferase cascade studied here is representative of its internal provision of uridine 5ʹ‐diphosphate (UDP)‐glucose from sucrose in the presence of catalytic amounts of UDP (Scheme 1) (Schmölzer et al., 2016). The UDP‐glucose is used for 3ʹ‐*β*‐*C*‐glycosylation of the flavonoid phloretin to yield nothofagin, a natural product *C*‐glycoside with strong antioxidative properties abundant in rooibos tea (Bungaruang et al., 2013).

Based on the synthetically relevant example of nothofagin, we have shown systematic engineering analysis of glycosyltransferase cascade reactions with the aim of achieving significant process intensification (Bungaruang et al., 2016; Schmölzer et al., 2018). In particular, using inclusion complexation with hydroxypropyl‐*β*‐cyclodextrin, solubility of the poorly water‐soluble phloretin (≤1 mM) was enhanced to approximately 100 mM without deteriorating the enzyme activity and stability, which the addition of organic co‐solvent (e.g., dimethyl sulfoxide) did. This enabled the complete conversion of phloretin for batch production of nothofagin (∼45 g/L) at a scale of approximately 100 g isolated product (Schmölzer et al., 2018). Considering the important transition from batch to continuous processing, we have in recent work shown the co‐immobilization of the glycosyltransferases for nothofagin production, that is, the *C*‐glycosyltransferase from rice (*Oryza sativa; Os*CGT) and the sucrose synthase from soybean (*Glycine max; Gm*SuSy) (Liu et al., 2021). Both enzymes were fused to the cationic binding module *Z*_basic2_ for their directed co‐immobilization on a porous carrier (Relisorb SP400 beads) harboring anionic (sulfonate) surface groups. The co‐immobilized *Os*CGT and *Gm*SuSy were approximately 70% as effective in nothofagin synthesis (Scheme 1) as the enzymes in solution. An essential component of their effectiveness was that both enzymes were co‐localized on the same carrier, which was 2.5‐fold superior for immobilization than immobilizing each enzyme on separate beads. The co‐immobilized enzymes were recycled over 15 batch reactions (Liu et al., 2021), but a fully continuous operation was not shown. Here we, therefore, developed a miniaturized packed‐bed reactor (1 ml volume) for continuous‐flow production of nothofagin by co‐immobilized *Os*CGT and *Gm*SuSy. Compared with the agitated vessel used previously, the packed bed involved a substantial (∼5‐fold) increase in solid catalyst loading (40 mg/ml → 200 mg/ml). This resulted in enhanced productivity (5.3‐fold; ∼11 mg/ml/h) due to residence time for full substrate conversion (60 mM phloretin) lowered to just approximately 2 h. We show continuous production of nothofagin (1.8 g; 90 ml) in 90 reactor cycles (2.3 h/cycle), reaching a total turnover number of up to 2.9 × 10^3^ mg product/mg immobilized enzyme used. Our study presents integrated process technology for flow synthesis with co‐immobilized sugar nucleotide‐dependent glycosyltransferases using efficient glycosylation from sucrose via the internally recycled UDP‐glucose.

## 2 MATERIALS AND METHODS

### 2.1 Materials

ReliSorb SP400 carrier was from Resindion S.R.L. 2‐Hydroxypropyl‐*β*‐cyclodextrin (>98%), phloretin (>98%), nothofagin (>98%), UDP (97%), and UDP‐glucose (>98%) were from Carbosynth. Unless indicated, all other chemicals were of analytical grade and obtained from Sigma‐Aldrich.

### 2.2 Enzymes

*N*‐terminal fusions of *Os*CGT and *Gm*SuSy with *Z*_basic2_ described in Liu et al. (2021) were used. The enzymes referred to as *Z*‐*Os*CGT and *Z*‐*Gm*SuSy (Figure S1; Table S1) were produced in *Escherichia coli* and purified by reported methods (Liu et al., 2021).

### 2.3 Enzyme immobilization

Before immobilization, the carrier (ReliSorb SP400; polymethacrylate particles of spherical shape, 75–200 μm diameter; 120 μm mean diameter; pore size ∼100 nm) was washed three times with water and two times with 4‐(2‐hydroxyethyl)‐1‐piperazineethanesulfonic acid (HEPES) buffer (50 mM, 250 mM NaCl, pH 7.5). Enzymes were immobilized directly from their *E. coli* cell lysates, obtained as reported in Liu et al. (2021). The immobilization via the *Z*_basic2_ module is fairly selective so that enzyme purification before immobilization is not necessary. Under the conditions used, other proteins were bound in only small amount (≤10%–15% of the total; see Section 3) and there was no evidence that they could have interfered with the enzyme performance (activity, stability, and loading). Briefly summarized, wet cells were suspended (1:1, by volume) in the above HEPES buffer and disintegrated by ultrasonication. The lysate was recovered by centrifugation (21,300 *g*, 4°C, 40 min; Centrifuge 5424R, Eppendorf). For co‐immobilization, the lysates were mixed to give the intended activity ratio. About 500 mg dry ReliSorb SP400 were incubated with lysate (5–12 ml; 4–12 mg protein/ml) at approximately 22°C (room temperature) on an end‐over‐end rotator at 40 rpm for 2 h. The beads were sedimented and washed three times with buffer. Enzyme activity and protein remaining in the supernatant (including the washing solutions) were measured and the immobilization yield was determined from the data. The activity of the immobilized enzyme(s) was also measured. The effectiveness factor of the immobilized enzymes was determined from the observable activity of the immobilized preparation (*V*_observable_,) and the activity bound on the carrier (*V*_bound_). The bound activity was determined from the activity balance in the solution. It is the difference between the activities in solution before and after the immobilization. The enzymes were stable in the time of the immobilization under the conditions used. Both *V*_observable_ and *V*_bound_ are expressed as U/g_carrier_. The ratio *V*_observable_/*V*_bound_ gives the effectiveness factor. The effectiveness factor was obtained for *Z*‐*Os*CGT and *Z*‐*Gm*SuSy individually as well as for the two enzymes working together in nothofagin synthesis. Protein or activity units on carrier (mg/g, U/g) are based on dry carrier mass.

### 2.4 Assays

Protein was measured with ROTI Quant reagent (Carl‐Roth) calibrated with bovine serum albumin (BSA). Reported assays were used for activity determination (Liu et al., 2021). The total liquid volume was 500 µl and incubation at 30°C with agitation (ThermoMixer C; Eppendorf) at 600 rpm (soluble enzymes) or 1000 rpm (immobilized enzymes). The sample (50 µl) taken at suitable times (typically 2 min) was mixed with 50 µl acetonitrile to stop the reaction. Solid material (precipitated protein and carrier beads) was centrifuged off at 13,200 rpm (Centrifuge 5424R, Eppendorf) for 20 min and the supernatant was analyzed by reversed‐phase high‐performance liquid chromatography (HPLC). One unit (U) is the enzyme amount releasing 1 µmol product/min under the conditions used. Full details of activity determination for the individual enzymes are shown in the Supporting Information (Table S2; Figure S2). The activity of the coupled glycosyltransferases was measured in HEPES buffer (50 mM, pH 7.5) containing 1.0 mM phloretin, 500 mM sucrose, 0.5 mM UDP, 50 mM KCl, 13 mM MgCl_2_, 1.3 mg/ml BSA, and 20% dimethyl sulfoxide (DMSO) as co‐solvent. HPLC analysis was done with a Kinetex® 5 µm EVO C18 LC column (100 Å, 150 × 4.6 mm; Phenomenex) using 20 mM potassium phosphate (pH 5.9) as the mobile phase (1 ml/min; 25°C). Elution was with a linear gradient of acetonitrile (25% → 60%) in 6 min. UV detection at 288 nm was used for the quantification of phloretin and nothofagin. For all activity measurements, it was ensured that the maximum conversion of the limiting substrate did not exceed 20%. Enzyme‐specific activities in the cell lysate and after purification by cation exchange chromatography, performed as reported in Liu et al. (2021), are summarized in Table S1.

### 2.5 Packed‐bed reactor

A Proteus FliQ FPLC Column (total volume, 1.0 cm^3^; diameter, 0.62 cm; height, 3.3 cm) from Protein Ark (Portobello) was used. Solid catalyst (∼200 mg ± 5%) was loaded into the column to cover the height fully, thus giving a total packed‐bed volume (*V*) of approximately 1.0 ml (±5%) (Figure S3). The total *V* (uncorrected for reactor porosity) was used to determine the residence time (τ_res_), according to τ_res_ = *V*/*F* where *F* is the liquid flow rate. Reactor porosity was estimated as approximately 0.8 or higher, with an assumed bed porosity of approximately 0.40 and a particle porosity of approximately 0.75 (dry matter content of wet particles; Bolivar et al., 2016). The reported τ_res_ may thus overestimate the actual residence time by maximally 20%. Conclusions of the study are unaffected by that.

Immobilized preparations of the individual enzymes were examined first. The co‐immobilized preparation was used for production. The column was placed in a water bath (30°C). Column inlet and outlet were connected with Teflon tubing (diameter, 250 µm; Micronit Microfluidics; Enschede, The Netherlands). A New Era NE‐1000 syringe pump (Next Advance) was used to deliver liquid flow in the range 0.003–0.50 ml/min. Before starting the reaction with substrate solution, the packed bed was washed with 25 ml of HEPES buffer (50 mM, pH 7.5) at 0.5 ml/min. The composition of the substrate and the flow conditions (residence times) are shown in Section 3. Unless mentioned, phloretin was used as inclusion complex with hydroxypropyl *β*‐cyclodextrin, prepared according to Liu et al. (2021). The molar ratio of phloretin and hydroxypropyl *β*‐cyclodextrin used was 1.25 and a stock solution of phloretin inclusion complex of 216 mM was prepared. The substrate solution was obtained by diluting the phloretin inclusion complex into the reaction buffer (500 mM sucrose, 0.5 mM UDP, 50 mM KCl, 13 mM MgCl_2_, 1.3 mg/ml BSA, 50 mM HEPES buffer, pH 7.5). This solution was prepared fresh before starting the experiment. The exact concentration of phloretin substrate used in the experiment was controlled by HPLC. Samples were taken at reactor outlet and analyzed by HPLC for nothofagin and phloretin, as described under Section 2.4. Optionally, the concentrations of UDP‐glucose and UDP were measured. This was done by ion‐pairing reversed phase HPLC using tetrabutylammonium bromide (40 mM) in phosphate buffer (20 mM, pH 5.9). Isocratic elution at 12.5% acetonitrile was used. Detection was at 262 nm (Figure S2b). For all reactions, close balance between substrate used and product formed was ensured. This also applied to UDP‐glucose and UDP. A shift in condition of continuous reaction (e.g., flow rate change; change in substrate concentration) was assessed only after five residence times, ensuring that the new steady state had been reached. Unless mentioned, the catalyst was prepared fresh but it was not changed in a related set of continuous experiments, such as those using a shift of the reaction condition. It was ensured that enzyme stability was sufficient. The solid catalyst was withdrawn from the column after continuous reaction over a longer time and analyzed by sodium dodecyl sulfate‐polyacrylamide gel electrophoresis (SDS‐PAGE) to detect possible elution of the adsorbed enzymes.

## 3 RESULTS AND DISCUSSION

### 3.1 Continuous reaction of the individual glycosyltransferases

**Figure 1:**
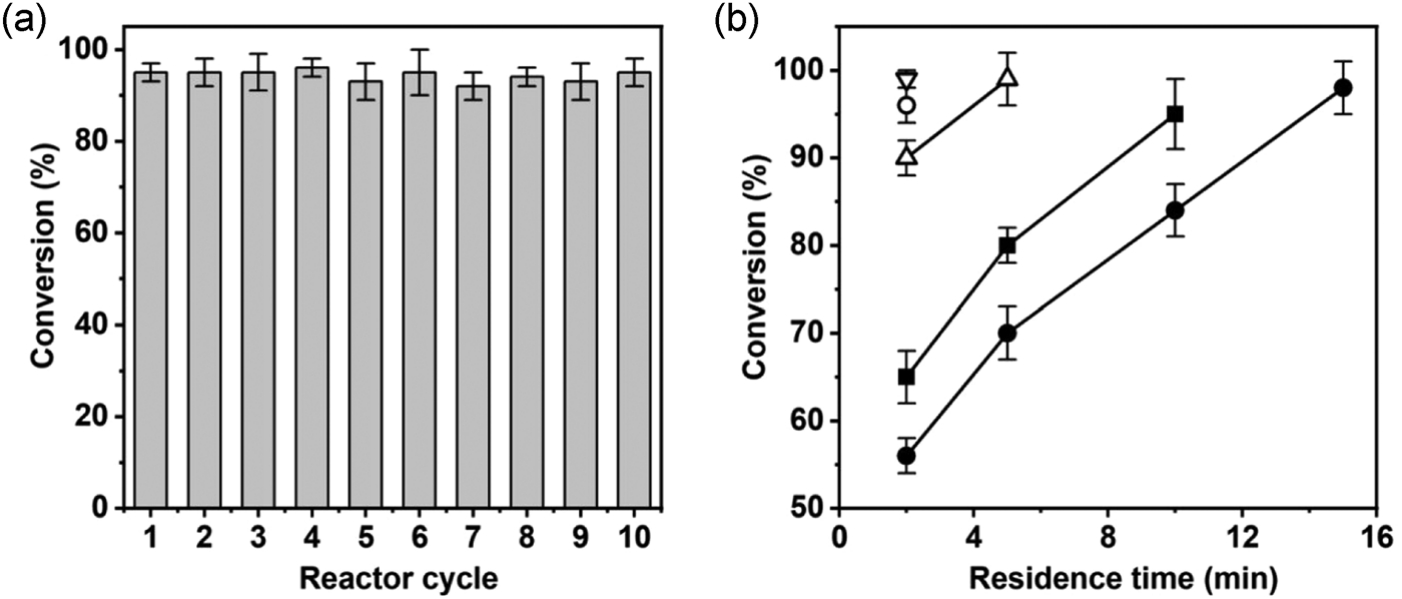
Assessment of individually immobilized glycosyltransferases (a, Z‐*Os*CGT; b, Z‐*Gm*SuSy) in continuous flow experiments. (a) Reaction of Z‐*Os*CGT at τ_res_ of 2 min. Conditions: 10 mM each of phloretin and UDP‐glucose, 50 mM KCl, 13 mM MgCl_2_, 50 mM HEPES buffer, pH 7.5. The solid enzyme activity was 19.5 U/g carrier (Table S2). (b) Reaction of Z‐*Gm*SuSy at varied τ_res_ and UDP concentration (in mM; ▽, 1.0; ○, 2.0; △, 5.0; ■, 8.0; ●, 10). Conditions: 500 mM sucrose, 50 mM Bistris buffer, pH 6.5. All reactions contained 50 mM KCl and 13 mM MgCl_2_. The solid enzyme activity was ∼20 U/g carrier (Table S2). *Gm*SuSy, sucrose synthase from soybean (*Glycine max*); HEPES, 4‐(2‐hydroxyethyl)‐1‐piperazineethanesulfonic acid; *Os*CGT, *C*‐glycosyltransferase from rice (*Oryza sativa*); UDP‐glucose, uridine 5ʹ‐diphosphate‐glucose

*Z*‐*Os*CGT (monomer; 57.8 kDa) and *Z*‐*Gm*SuSy (homotetramer; subunit 100.7 kDa) were immobilized separately on the ReliSorb SP400 carrier (Table S2) and assessed for activity in continuous operation. The idea was to start by examining the individual glucosyl transfers of the overall cascade glycosylation (Scheme 1) as isolated reaction steps. Figure 1a shows the reaction of *Z*‐*Os*CGT at a constant τ_res_ of 2 min, using 10 mM of each UDP‐glucose and phloretin. The substrate conversion was approximately 95% (±5%) and stable over 10 reactor cycles. The observed conversion rate (4.5 mM/min) was in useful agreement with expectation (~4 mM/min) from the immobilized *Z*‐*Os*CGT activity (~20 U/g dry carrier, Table S2). Figure 1b shows the reaction of *Z*‐*Gm*SuSy, analyzed for conversion of the limiting UDP substrate (used at variable concentrations between 1.0 and 10 mM) dependent on the τ_res_. The resulting dependencies (e.g., that of 10 mM UDP) were curves, featuring a pronounced slowing down of the glycosylation rate at high conversion (≥50%) due to substrate depletion, product accumulation, or both. Although the immobilized *Z*‐*Gm*SuSy activity was comparable to that of *Z*‐*Os*CGT, the τ_res_ for complete (≥ 95%) conversion of 10 mM UDP was 7.5‐fold larger (15 min). It is worth noting that reaction coupling (Scheme 1) can mitigate the slowdown of the rate, due to its combined effect of lowered product inhibition and maintained supply of substrate. Using the results at small τ_res_ and low conversion (Figure 1b; 10 mM UDP), a maximum rate of 2.8 mM/min was determined for the packed bed of immobilized *Z*‐*Gm*SuSy. From the immobilized enzyme activity measured in well‐mixed suspension (Table S2; 20 U/g dry carrier), a rate of approximately 4 mM/min was expected. This suggested a decrease by approximately 30% in the effectiveness factor of the immobilized *Z*‐*Gm*SuSy in the packed bed reactor. External (liquid‐solid) mass transport might be more relevantly limiting for the overall reaction rate in a packed bed as compared to a well‐mixed suspension of particles. Repulsion of like (negative) charges on the UDP and the carrier could be an important factor of the mass transfer rate (Blanch & Clark, 1996). We return to this point later when discussing the coupled enzyme reaction.

### 3.2 Enzyme co‐immobilization

**Figure 2:**
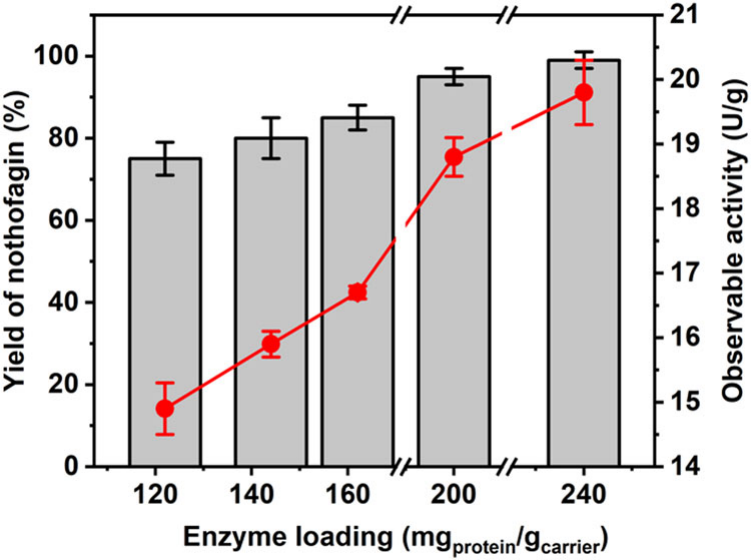
Co‐immobilization of Z‐*Os*CGT and Z‐*Gm*SuSy for reaction in continuous flow. The nothofagin yield from continuous reaction (gray bars) and the activity from batch assays (red circles) for solid enzyme preparations with varied enzyme loading (Table S3) are shown. Conditions: 10 mM phloretin, 500 mM sucrose, 0.5 mM UDP, 50 mM KCl, 13 mM MgCl_2_, 1.3 mg/ml BSA, all in 50 mM HEPES buffer, pH 7.5. The τ_res_ was 10 min. For the conditions of the batch assay, see Section 2. BSA, bovine serum albumin; *Gm*SuSy, sucrose synthase from soybean (*Glycine max*); HEPES, 4‐(2‐hydroxyethyl)‐1‐piperazineethanesulfonic acid; *Os*CGT, *C*‐glycosyltransferase from rice (*Oryza sativa*); UDP, uridine 5ʹ‐diphosphate

Based on Liu et al. (2021), who showed that a *Z*‐*Os*CGT/*Z*‐*Gm*SuSy activity ratio of approximately 1.2 in the loaded mixture of cell lysates was optimal for the overall synthetic activity of the co‐immobilized enzyme preparation, we here aimed at enhancing the immobilized nothofagin activity (U/g carrier) while keeping the loaded enzyme activity ratio constant. Individual immobilization of *Z*‐*Os*CGT and *Z*‐*Gm*SuSy on Relisorb SP400 was studied in detail before (Liu et al., 2021) and the enzymes shown to give fairly similar results. Although *Gm*SuSy is a homotetramer that could use multivalency from four *Z*_basic2_ modules for binding, the immobilization of *Z*‐*Gm*SuSy is not much stronger than that of *Z*‐*Os*CGT. Liu et al. (2021) discuss the results in relation to structural features of the sucrose synthase. Here, total protein loading was varied between 122 mg/g and 240 mg/g and the immobilization results are summarized in Table S3. The nothofagin activity increased roughly linearly with total protein loading, as shown in Figure 2. When loading 240 mg protein/g carrier, *Z*‐*Os*CGT and *Z*‐*Gm*SuSy were co‐immobilized at an individual activity of 20.5 U/g and 18.9 U/g, respectively. The combined (overall) activity of nothofagin synthesis was 19.8 U/g. The immobilization yield and the effectiveness factor of each enzyme decreased from approximately 90% to 70% as the loading increased from 122 mg/g to 240 mg/g (Table S3). Compared with the earlier study (Liu et al., 2021), a 1.5‐fold increase in co‐immobilized enzyme activity (nothofagin synthesis rate) was achieved. While it may be possible to still increase the enzyme loading further, this will probably be achievable only at the expense of the immobilization yield and the effectiveness factor.

### 3.3 Continuous reaction of co‐immobilized glycosyltransferases

**Figure 3:**
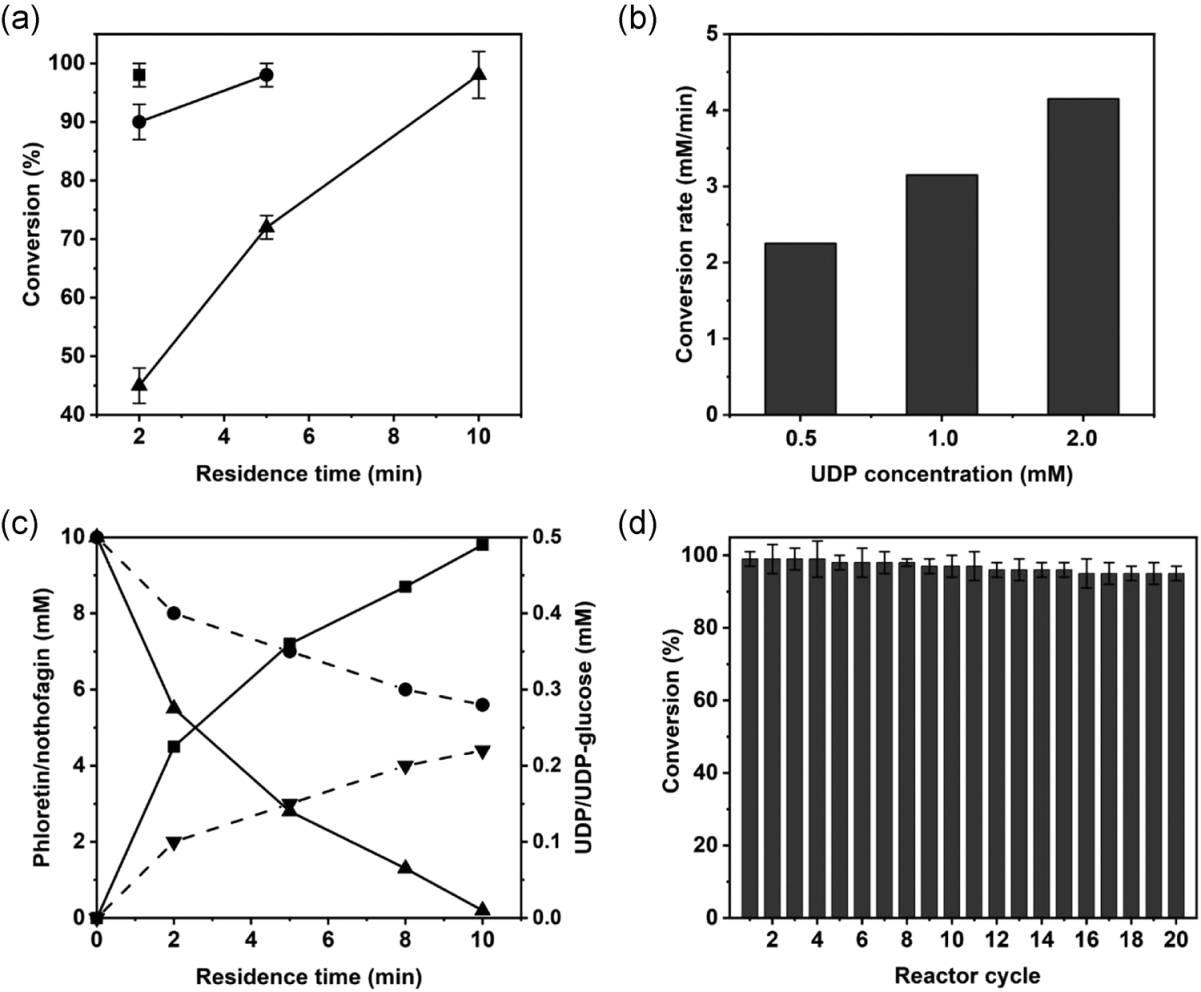
Evaluation of co‐immobilized Z‐*Os*CGT and Z‐*Gm*SuSy in terms of efficiency and stability. (a) Reaction at different concentrations of phloretin (mM; ■, 1.0; ●, 5.0; ▲, 10) at varied τ_res_. (b) Reaction at different concentrations of UDP (mM; 0.5; 1.0; 2.0) using 10 mM phloretin at τ_res_ of 2 min. (c) Steady‐state reactant concentrations (■, nothofagin; ▲, phloretin; ●, UDP; ▼, UDP‐glucose) for conversion of phloretin (10 mM) at varied τ_res_. (d) Continous conversion of phloretin (10 mM) at τ_res_ of 10 min. Conditions: 500 mM sucrose, 0.5 mM UDP, 50 mM KCl, 13 mM MgCl_2_, 1.3 mg/ml BSA, 50 mM HEPES buffer (pH 7.5); 30°C. In all reactions, solid enzyme preparation with 19.8 U/g carrier (Table S3) was used. BSA, bovine serum albumin; *Gm*SuSy, sucrose synthase from soybean (*Glycine max*); HEPES, 4‐(2‐hydroxyethyl)‐1‐piperazineethanesulfonic acid; *Os*CGT, *C*‐glycosyltransferase from rice (*Oryza sativa*); UDP, uridine 5ʹ‐diphosphate

Figure 2 shows the continuous conversion of 10 mM phloretin using the co‐immobilized enzyme preparations indicated. With τ_res_ at 10 min, the yield of nothofagin increased dependent on the immobilized activity. Further experiments were therefore performed with the most active preparation (~20 U/g). Figure 3a shows the conversion of phloretin, applied at variable concentrations in the range 1.0‐10 mM, dependent on τ_res_. From results at low conversion (10 mM phloretin; τ_res_ = 2 min), we calculated a reaction rate of 2.25 mM/min. From the immobilized activity, a rate of approximately 4 mM/min was expected. This suggested that the co‐immobilized enzyme used in the packed bed reactor was only 56% as active as the same enzyme preparation used in the well‐mixed suspension of particles. Order of magnitude estimate for external mass transfer in the packed bed (Online Supporting Information) gave a transport coefficient (*k*_L_ *a*) of approximately 21 min^−1^. With this *k*_L_ *a*, the Damköhler number (*Da* = *r*/*k*_L_ *a* [*S*]_b_) was estimated as 0.21. [*S*]_b_ is the limiting substrate concentration (0.5 mM UDP) and *r* is the reaction rate (2.25 mM/min). The *K*_M_ of *Gm*SuSy for UDP is 0.13 mM and the coupled reaction of *Os*CGT and *Gm*SuSy in solution was saturated at 0.5 mM UDP (Bungaruang et al., 2013). With these numbers (*Da* = 0.21; [*S*]_b_/*K*_M_ = 3.85), a substantial decrease in immobilized enzyme effectiveness due to external transport limitation under conditions of the flow reactor is not plausibly explained (for the general case, see Doran, 2016). Uncertainty in the estimation of *k*_L_ *a*, especially under the possible involvement of charge repulsion between the solute and the solid surface, however, precluded a more quantitative analysis of the effect in terms of the transport rate. Nevertheless, continuous experiments performed at varied UDP concentrations (0.5–2.0 mM) clarified the implied limitation of the overall conversion rate by the UDP available to the immobilized enzymes (Z‐*Gm*SuSy). Figure 3b shows that increased usage of UDP gave an enhanced conversion rate. Compared with the 2.25 mM/min at 0.5 mM UDP, the conversion rate was increased 1.4‐fold (3.15 mM/min) at 1.0 mM UDP. At 2.0 mM UDP, it was 4.15 mM/min and so approached the rate expected from the activity measurements done in mixed suspension (Table S3).

Figure 3c shows the steady‐state concentrations of UDP and UDP‐glucose dependent on τ_res_ as the conversion of phloretin (10 mM) into nothofagin progressed. At low τ_res_, UDP was present in four‐fold excess over UDP‐glucose, indicating UDP‐glucose consumption (*Z*‐*Os*CGT reaction) faster than formation (*Z*‐*Gm*SuSy reaction). The activities of immobilized *Z*‐*Os*CGT and *Z*‐*Gm*SuSy were almost identical, as shown in Table S3. The above notion, that the overall conversion rate was limited by the supply of UDP to the *Z*‐*Gm*SuSy reaction, was therefore strongly supported. Figure 3c further shows that the UDP‐glucose increased with increasing τ_res_ to become similar to the UDP concentration when the phloretin conversion was almost complete. The trend was consistent with the idea that, contrary to the *Z*‐*Os*CGT reaction that was likely slowed down under conditions of phloretin depletion and nothofagin accumulation, the *Z*‐*Gm*SuSy reaction would not be similarly affected due to sucrose used in large (50‐fold) surplus over phloretin.

Figure 3d shows continuous reaction (10 mM phloretin) performed over 20 reactor cycles. The substrate conversion (≥95%) was maintained, indicating excellent stability of the co‐immobilized enzyme preparation under conditions of use. The packed‐bed flow reactor operated at a steady state was a practical engineering tool to examine factors of enzyme activity and stability during the reaction. DMSO co‐solvent (20%, by volume) only moderately decreased the overall activity of the coupled glycosyltransferases by approximately 15%, as shown in Figure S4 that compares the dependence on τ_res_ for the conversion of phloretin (10 mM) solubilized with DMSO or hydroxypropyl *β*‐cyclodextrin. More importantly, however, the DMSO caused substantial decrease in the enzyme stability (Figure S4). Continuous reaction performed as in Figure 3c showed rapid decrease in phloretin conversion from 95% initially to approximately 50% after 20 reactor cycles (Figure S4b). Interestingly, the decrease in conversion was associated with an increase in the UDP concentration from 0.25 mM (as in Figure 3b at high τ_res_) to 0.38 mM, suggesting that UDP‐glucose formation (*Z*‐*Gm*SuSy reaction) was more strongly affected than UDP‐glucose consumption (*Z*‐*Os*CGT reaction). Analyses done on the solid catalyst recovered from the continuous reaction revealed (Figure S4e) that activity loss was indeed more pronounced for *Z*‐*Gm*SuSy (31%) than *Z*‐*Os*CGT (19%). Desorption of enzyme from the solid carrier appeared not to be a major factor of catalyst stability, as suggested by SDS‐PAGE (Figure S4d) showing similarly strong protein bands of *Z*‐*Gm*SuSy and *Z*‐*Os*CGT at reaction start and end. The aggregate data from the continuous reaction in the presence of DMSO co‐solvent shows that the positive effect of using phloretin as inclusion complex with *β*‐cyclodextrin goes beyond solubility enhancement of the acceptor substrate. It involves efficient use of the stabilized enzyme activity additionally, as shown by comparing Figures 3d with S4. In a separate set of experiments, we examined the role of added salts (i.e., NaCl and MgCl_2_) on coupled glycosyltransferase activity. Figure S5 shows that the supplementation of salts was important for full enzyme activity in the continuous reaction.

### 3.4 Continuous production of nothofagin

**Figure 4:**
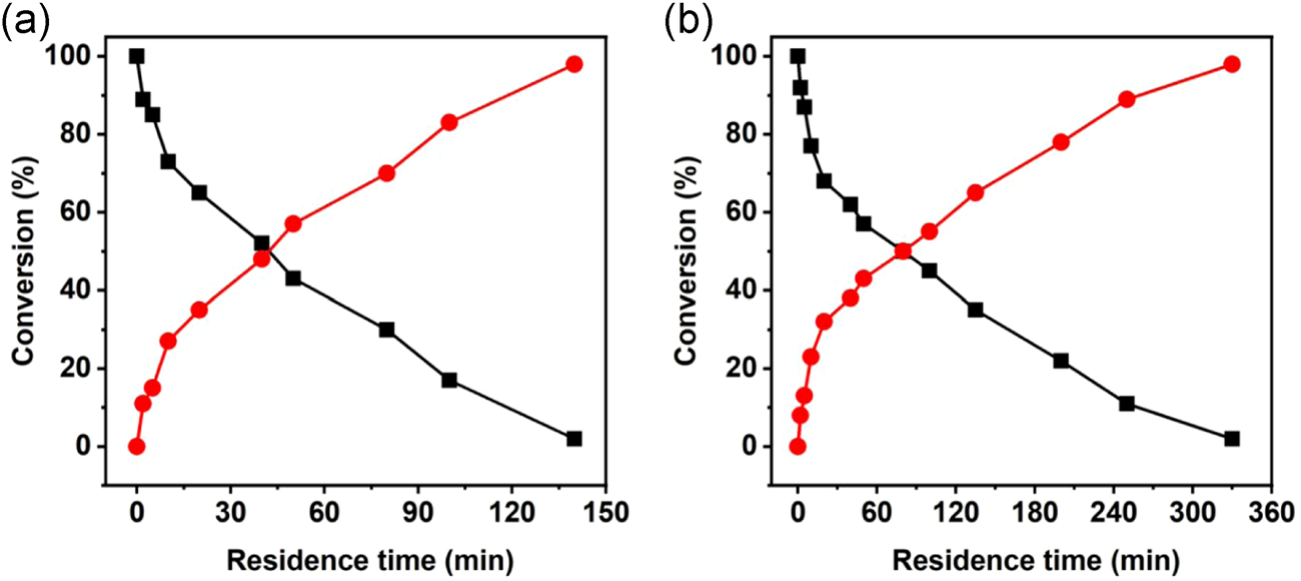
Flow synthesis of nothofagin (circles, red) from phloretin (squares, black; a, 60 mM; b, 120 mM) using co‐immobilized Z‐*Os*CGT and Z‐*Gm*SuSy. Conditions: 500 mM sucrose, 0.5 mM UDP, 50 mM KCl, 13 mM MgCl_2_, 1.3 mg/ml BSA, 50 mM HEPES buffer (pH 7.5); 30°C. In all reactions, solid enzyme preparation with 19.8 U/g carrier (Table S3) was used. The enzyme preparation was the same in each series of residence time change. It was changed between the experiment in panel (a) and panel (b). It was ensured that enzyme stability was sufficient in each series. BSA, bovine serum albumin; *Gm*SuSy, sucrose synthase from soybean (*Glycine max*); HEPES, 4‐(2‐hydroxyethyl)‐1‐piperazineethanesulfonic acid; *Os*CGT, *C*‐glycosyltransferase from rice (*Oryza sativa*); UDP, uridine 5ʹ‐diphosphate

We analyzed continuous reaction at high phloretin concentration (60 mM; 16.4 g/L) suitable for nothofagin production. Systematic variation of the τ_res_ revealed that using a catalyst with approximately 20 U/g carriers, about 140 min were necessary to achieve complete conversion of the phloretin (Figure 4a). The phloretin concentration was increased to 120 mM and full conversion was shown as well, although the required τ_res_ was more than doubled (2.4‐fold; Figure 4b). Experiments at lower phloretin concentration (20–50 mM; Figure S6) showed that the τ_res_ for full conversion scaled proportionally with the substrate concentration used. Working in agitated vessel in batch reaction, Schmölzer et al. (2018) noted viscous fluid mixing to become a physical boundary of the conversion of concentrated solutions (150 mM) of the phloretin inclusion complex, with sucrose (500 mM) additionally present.

**Figure 5:**
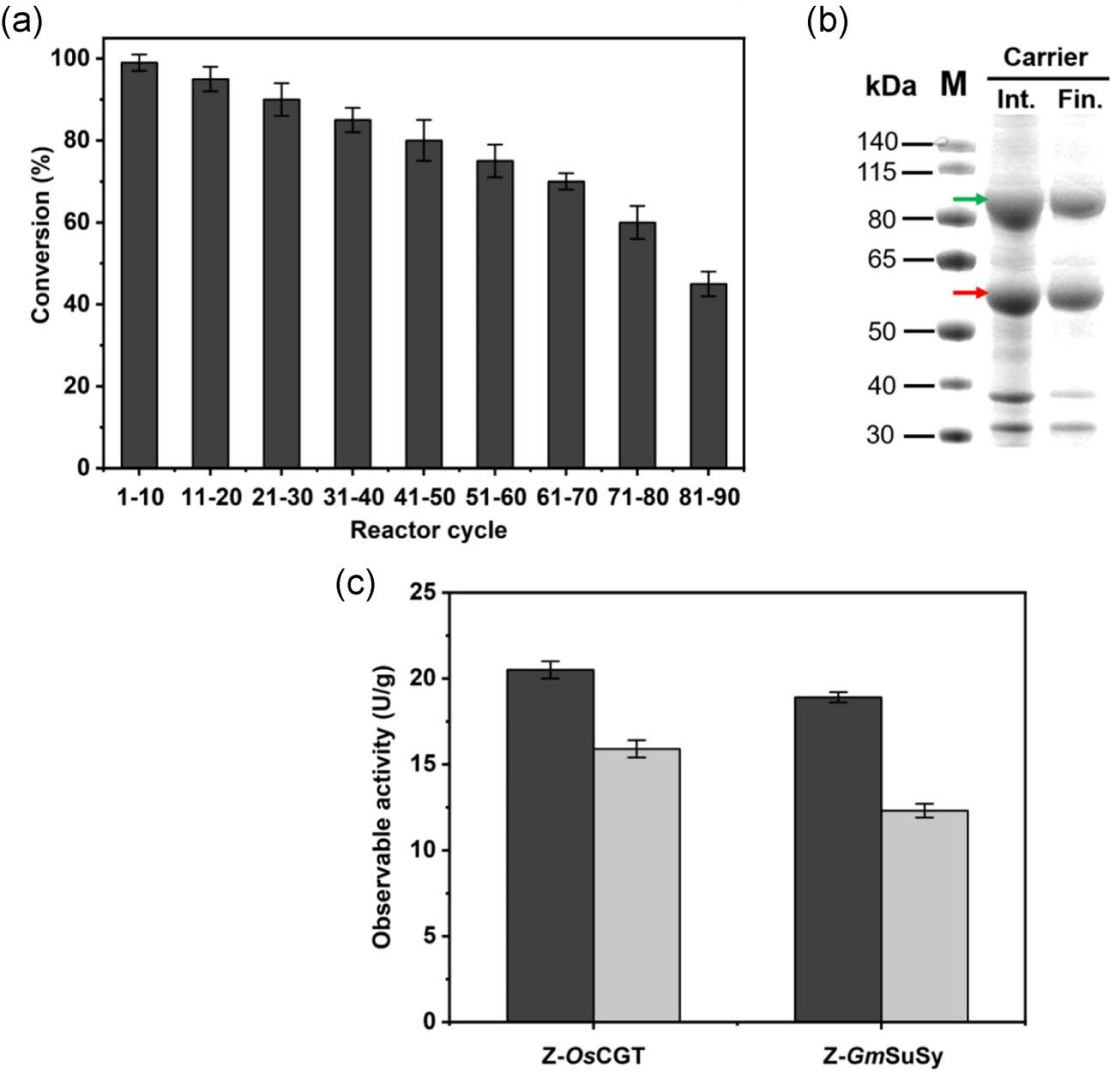
Continuous conversion of phloretin with co‐immobilized Z‐*Os*CGT and Z‐*Gm*SuSy. (a) Reaction with 60 mM phloretin at τ_res_ of 140 min. Conditions: 500 mM sucrose, 0.5 mM UDP, 50 mM KCl, 13 mM MgCl_2_, 1.3 mg/ml BSA, 50 mM HEPES buffer (pH 7.5); 30°C. In all reactions, solid enzyme preparation with 19.8 U/g carrier (Table S3) was used. (b) SDS polyacrylamide gel showing the protein on the ReliSorb SP400 carrier at reaction start (Int) and after the last reaction (Fin). About 10 mg of wet carrier was suspended directly in 20 µl SDS loading buffer. After boiling for 10 min, the supernatant was loaded on the gel. (c) Immobilized Z‐*Os*CGT and Z‐*Gm*SuSy activity at reaction start (black bars) and after 90 reaction cycles (gray bars). The wet carrier (20 mg) was diluted into the reaction mixture and the assay for the individual enzyme activity performed as shown in Table S2. BSA, bovine serum albumin; *Gm*SuSy, sucrose synthase from soybean (*Glycine max*); HEPES, 4‐(2‐hydroxyethyl)‐1‐piperazineethanesulfonic acid; *Os*CGT, *C*‐glycosyltransferase from rice (*Oryza sativa*); SDS, sodium dodecyl sulfate; UDP, uridine 5ʹ‐diphosphate

To perform continuous reaction over a longer time at manageable viscosity, we, therefore, chose 60 mM phloretin (τ_res_ = 140 min) and show nothofagin production over 90 reactor cycles (Figure 5a). The pooled product solution (90 ml) contained 1.8 g nothofagin released in 210 h. The phloretin conversion decreased gradually, indicating that enzyme activity (∼45%) was lost during the continuous reaction. The product solution therefore contained 0.5 g unreacted phloretin. The catalyst half‐life of approximately 200 h under in operando conditions of the flow reactor (Figure 5a) was consistent with evidence from an earlier study (Liu et al., 2021) that used recycling of co‐immobilized *Z*‐*Os*CGT and *Z*‐*Gm*SuSy in repeated batch reaction. After 15 cycles of 12 h batch reaction (180 h) about half of the catalyst activity was lost (Liu et al., 2021). Here, the solid catalyst was recovered at the end of the continuous reaction and analyzed by SDS‐PAGE (Figure 5b). Both *Z*‐*Os*CGT and *Z*‐*Gm*SuSy were still bound to the carrier, but the immobilized amount of each enzyme was decreased substantially compared to the beginning of the reaction. To quantitate the overall activity loss, we measured the individual enzyme activities (Figure 5c) and showed substantial decrease of both (*Z*‐*Os*CGT: 22%; *Z*‐*Gm*SuSy: 35%). Since the overall activity of the catalyst depends not only on the activity of the individual glycosyltransferases but also on the activity ratio of the two (Liu et al., 2021), the observed decrease in phloretin conversion could arguably be explained by the data (Figure 5c). Experiments in the presence of DMSO co‐solvent (Figure S4d) suggested that enzymes still bound to the carrier had lost their activity, implying that enzyme inactivation not necessarily involved desorption from the solid support. Tentatively, therefore, we propose enzyme lixiviation as main reason for the observed decrease in productivity of the catalytic flow reactor under conditions when inclusion complexation with hydroxypropyl *β*‐cyclodextrin was used for solubilization of the acceptor substrate. Compared to the soluble enzymes (lacking the *Z*_basic2_ module) that showed activity half lives of approximately 20 h (Bungaruang et al., 2013), the in‐operando stability of the co‐immobilized *Z*‐*Os*CGT and *Z*‐*Gm*SuSy was enhanced by at least one order of magnitude.

From these results (Figure 5), key parameters of the process performance are the following. The volumetric productivity (≥95% phloretin conversion) was approximately 11 mg/(ml/h) over the first 10 reaction cycles. With the 1 ml working volume used, the mass productivity was therefore approximately 11 mg/h. The catalyst productivity was 1.9 h^−1^ and 0.055 h^−1^ when based on enzyme mass and total mass of solid catalyst, respectively. Note that the enzyme accounted for only approximately 3% of the solid mass. The enzyme total turnover number (*TTN*) was estimated from the portion of individual activity lost in the process and the known amount of enzyme immobilized. The Supporting Information shows the calculations. For Z‐*Os*CGT, the *TTN* was 2.9 × 10^3^ mg/mg. For Z‐*Gm*SuSy, it was 1.8 × 10^3^ mg/mg. On a mole basis (*M*_r_ nothofagin: 436.4; *M*_r_ Z‐*Os*CGT: 57849; *M*_r_ Z‐*Gm*SuSy (subunit): 100661), the *TTN* was 3.8–4.1 × 10^5^. These enzyme *TTN* values are excellent from a global perspective in applied bio‐catalysis (Sheldon & Woodley, 2018) but they are truly outstanding for Leloir glycosyltransferases. The few reported *TTN* values for glycosyltransferases applied to natural product glycosylation are typically in the single‐digit g/g range (e.g., Trobo‐Maseda et al., 2020; for review, see Nidetzky et al., 2018). Limitations arise from low acceptor substrate concentration soluble in reaction medium as well as from low enzyme activity and stability. Lack of enzyme recycling is another limitation. Using glycosyltransferases for oligosaccharide synthesis in which the issue of substrate solubility does not arise in general, the *TTN* values are higher (≤10^3^ g/g), as might be expected (for reviews, see: Nidetzky et al., 2018; Schelch et al., 2020). For batch synthesis of UDP‐glucose from sucrose and UDP by *Gm*SuSy, a *TTN* of 1440 was obtained (Gutmann & Nidetzky, 2016; Schmölzer et al., 2017). Optimized oligosaccharide synthesis by multienzyme cascades gave *TTN* values in the range 2.1 × 10^4^–7.2 × 10^5^ (Tsai et al., 2013). Important advance of the current study was to demonstrate cascade glycosylation with co‐immobilized glycosyltransferases in a continuous packed‐bed reactor and to show the reaction intensification thus obtainable. The flow synthesis thus realized combines high enzyme *TTN* with excellent performance metrics of the catalytic reaction, namely product yield, product concentration and *STY*. The observed decrease in conversion (∼50% in 210 h; Figure 5a) could be compensated by suitable adjustment of the residence time every 10 or 20 cycles. To avoid decrease in reactor productivity resulting from the required increase in τ_res_, an alternative would be to load fresh enzyme on the reactor while in operation. Although not pursued in the current study but shown with other enzymes before (Bolivar, Krämer et al., 2016; Bolivar, Tribulato et al., 2016; Bolivar et al., 2017; Valikhani, Bolivar, Viefhues et al., 2017; Valikhani, Bolivar, Pfeiffer et al., 2017; Valikhani et al., 2020), the protein surface tethering via the *Z*_basic2_ module is suitable for selective enzyme immobilization in flow, using facile loading procedure.

## 4 CONCLUSIONS

This study of nothofagin synthesis from phloretin and sucrose in the presence of catalytic amounts of UDP (0.83 mol%) demonstrates, for the first time, a two‐enzyme glycosyltransferase cascade co‐immobilized on solid support for fully continuous production. Reaction in packed‐bed flow reactor involved excellent metrics in terms of *TTN, STY*, substrate conversion, and product concentration. The good *TTN* was the combined result of efficient processing in terms of *STY* and enzyme recycling and stabilization due solid co‐immobilization. Processing in fully continuous operation compared with processing (repeated) batch processing (Liu et al., 2021; Schmölzer et al., 2018) was more efficient (∼5‐fold) proportionally to the increased enzyme loading/reactor working volume used. This was consistent with expectation, based on fundamental engineering principles, of no *intrinsic* process intensification due to change from well mixed batch reactor to continuous tubular (plug‐flow) reactor. Overall, a modular process technology for biocatalytic glycosylation from sucrose via UDP‐glucose was suggested. Being new to the field of Leloir glycosyltransferases, it might stimulate the development of more of these extremely versatile and powerful enzymes for scalable production of natural product glycosides and oligosaccharides.

## AUTHOR CONTRIBUTIONS

Hui Liu and Bernd Nidetzky designed the research; Hui Liu performed experiments and analyzed data. Bernd Nidetzky and Hui Liu wrote the paper.

## Data Availability

The data that support the findings of this study are available from the corresponding author upon reasonable request.
