# Supplementary material for: Leloir glycosyltransferases enabled to flow synthesis: Continuous production of the natural C‐glycoside nothofagin
Source: Biotechnol Bioeng. 2021 Aug 16;118(11):4402–13. doi: 10.1002/bit.27908 (PMC9291316; doi:10.1002/bit.27908)
Supplement: Supplementary file 1 [file BIT-118-4402-s001.docx]

**SUPPORTING INFORMATION**

**Leloir glycosyltransferases enabled to flow synthesis: continuous production of the natural *C*-glycoside nothofagin**

Hui LIU^1^ and Bernd NIDETZKY^1,2,*^

^1^ Institute of Biotechnology and Biochemical Engineering, Graz University of Technology, NAWI Graz, A-8010 Graz, Austria

^2^ Austrian Centre of Industrial Biotechnology (acib), A-8010 Graz, Austria

* Corresponding author (B.N.); e-mail, bernd.nidetzky@tugraz.at; phone: +433168738400

**S1. Characterization of fluid flow and external liquid-solid mass transfer**

Average diameter of particle (*d*_p_): 1.2 × 10^-4^ m

External porosity of the packed bed (ε): ~0.4 (assumed from literature)^[a]^

Surface area (*a*, m^-1^): *a* = 6(1 – ε)/*d*_p_ = 3 × 10^4^ m^-1^

Diffusion coefficient^[b]^ (*D*, 2-hydroxypropyl-β-cyclodextrin): 1.8 × 10^-8^ m^2^ min^-1^

Kinematic viscosity^[c]^ (*v*, 500 mM sucrose): 7.62 × 10^-5^ m^2^ min^-1^

Axial flow rate (*v*_ax_): 1.65 × 10^-2^ m min^-1^

Reynolds number (*Re*): *Re* = *v*_ax_ *d*_p_/ε*v* = 0.065

The *Re* number far below 1 implies laminar flow.

Sherwood number (*Sh*): *Sh* = *k*_L_*d*_p_/*D*, where is liquid-side mass transfer coefficient (m min^-1^)

For laminar flow conditions, we have^[d]^

*Sh* = 0.99 *Re*^1/3^ *Sc*^1/3^, where *Sc* is Schmidt number (*Sc* = *v*/*D* = 4233).

It follows that:

*Sh* = 0.99 (*v*_ax_ *d*_p_/ε*D*)^1/3^ = 6.50

From the known *Sh*, we obtain the estimate of *k*_L_ as:

*k*_L_ = 9.8 × 10^-4^ m min^-1^

Therefore, *k*_L_*a* = 29.4 min^-1^

An alternative correlation (*Frössling correlation*) for the Sherwood number *Sh* is,^[e]^

*Sh* = 2 + 0.6 *Re*^1/2^ *Sc*^1/3^ = 4.74

Thus, the corresponding estimate of the *k*_L_ is 7.1 × 10^-4^ m min^-1^.

The *k*_L_*a* is 21.3 min^-1^.

^[a]^ Nemec D., Levec J. *Chem. Eng. Sci.* 2005, **60**, 6947-6957

^[b]^ Ribeiro A.C.F. et al. *J. Chem. Eng. Data* 2007, **52**, 586-590. The *D* value (0.307 × 10^-9^

m^2^ s^-1^) from Table 3 for 0.010 M 2-hydroxypropyl-β-cyclodextrin at 298.15 K is used.

^[c]^ Simion A.I. et al. *Annals Food Sci. Technol.* 2011, **2**, 225-232

^[d]^ Muttzall K. Modellierung von Bioprozessen. 1995, Behr’s Verlag, Hamburg; p. 200

^[e]^ Fogler H. S. Elements of Chemical Reaction Engineering (6^th^ Edition). 2020. Pearson, London, UK. Chapter 14, pp. 739-790.

**S2. Calculation of total turnover numbers (*TTN*)**

The *TTN* is expressed as the ratio of mass or mol product formed and the mass or mol enzyme protein used (inactivated) in the reaction (Δ*E*_mass,_ Δ*E*_mol_). The total amount of nothofagin produced in 90 reaction cycles was 1.8 g (*P*_mass_) or 4.1 mmol (*P*_mol_). Therefore,

*TTN*_mass_ = *P*_mass_/Δ*E*_mass_

*TTN*_mol_ = *P*_mol_/Δ*E*_mol_

The enzyme used was calculated from the protein amount immobilized and the percent activity lost in the reaction. The protein amount immobilized was calculated from the activity amount immobilized and the specific activity of the purified enzyme. The activity amount immobilized was calculated from the activity offered in the immobilization and the activity remaining in solution after immobilization. The table below summarized the calculations.

| Enzyme | Activity immobilized  (U/g carrier) | Enzyme protein immobilized  (mg/g carrier) | Percent activity lost  (%) | Enzyme protein consumed^[a]^ |
| --- | --- | --- | --- | --- |
| Z-*Os*CGT | 32 | 14.0 | 22 | 0.62 |
| Z-*Gm*SuSy | 27 | 14.3 | 35 | 1.00 |

^[a]^In a 1 ml packed bed reactor containing 0.20 g solid material.

With these data, the *TTN*_mass_ and *TTN*_mol_ of the individual enzymes were calculated.

| Enzyme | *TTN*_mass_ | *TTN*_mol_ |
| --- | --- | --- |
| Z-*Os*CGT^[a]^ | 2.9 × 10^3^ | 3.8 × 10^5^ |
| Z-*Gm*SuSy^[b]^ | 1.8 × 10^3^ | 4.1 × 10^5^ |

^[a]^ The molecular weight of Z-*Os*CGT is 57849.

^[b]^ The molecular weight of Z-*Gm*SuSy is 100661.

**SUPPORTING TABLES**

**Table S1.** Specific activities of Z-*Os*CGT and Z-*Gm*SuSy

| **Enzyme** | **U/mg**^[a]^ | **U/mg**^[b]^ |
| --- | --- | --- |
| Z-*Os*CGT^[c]^ | 0.36 ± 0.05 | 2.28 ± 0.05 |
| Z-*Gm*SuSy^[d]^ | 0.30 ± 0.04 | 1.89 ± 0.03 |

^[a]^ *E. coli* cell lysate

^[b]^ Purified enzyme

^[c]^ Assay: 1.0 mM phloretin, 2.0 mM UDP-glucose, 50 mM KCl, 13 mM MgCl_2_, 4% DMSO, 50 mM HEPES buffer (pH 7.5); 30°C; analysis by HPLC for nothofagin formed (Figure S2A).

^[c]^ Assay: 500 mM sucrose, 1.0 mM UDP, 50 mM KCl, 13 mM MgCl_2_; 50 mM BisTris buffer (pH 6.5); 30°C; analysis by HPLC for UDP-glucose formed (Figure S2B).

Mean values of 4 independent determinations and the corresponding standard errors are shown.

**Table S2.** Immobilization of individual enzymes.

| **Enzyme**^[a]^ | **Enzyme loading (mg_protein_/g_carrier_)** | **Observable activity (U/g)** | **Immobilized yield (%)** | **Catalytic effectiveness (%)** |
| --- | --- | --- | --- | --- |
| Z-*Os*CGT^[b]^ | 84 (30)^[d]^ | 18.4 ± 0.4 | 74 ± 2 | 83 ± 4 |
|  | 112 (40)^[d]^ | 19.5 ± 0.5 | 66 ± 3 | 73 ± 3 |
| Z-*Gm*SuSy^[c]^ | 91 (27)^[d]^ | 17.6 ± 0.1 | 80 ± 5 | 77 ± 4 |
|  | 126 (38)^[d]^ | 20.0 ± 0.4 | 75 ± 2 | 71 ± 2 |

^[a]^ Z-*Os*CGT and Z-*Gm*SuSy were immobilized on the Relisorb SP400.

^[b]^ Assay: 1.0 mM phloretin, 2.0 mM UDP-glucose, 50 mM KCl, 13 mM MgCl_2_, 4% DMSO, 50 mM HEPES buffer (pH 7.5); 30°C; analysis by HPLC for nothofagin formed.

^[c]^ Reaction conditions: 500 mM sucrose, 1.0 mM UDP, 50 mM KCl, 13 mM MgCl_2_; 50 mM BisTris buffer (pH 6.5); 30°C; analysis by HPLC for UDP-glucose formed.

^[d]^ The value in brackets is the loaded U/g carrier.

Mean values of three independent determinations and the corresponding standard errors are shown.

**Table S3.** Co-immobilization of Z-*Os*CGT and Z-*Gm*SuSy on ReliSorb SP400.

| **Ratio enzyme loading**^[a]^ | **Enzyme loading (mg _protein_/g _carrier_)**^[b]^ | **Observable activity (U/g)**^[c]^ | **Protein** | **Individual enzyme loading (U/g**_c_**_arrier_)** | **Individual enzyme observable activity (U/g)^[^**^d]^ | **Immobilized yield (%)** | **Catalytic effectiveness (%)** |
| --- | --- | --- | --- | --- | --- | --- | --- |
| 1.2 | 122 | 14.9 ± 0.4 | *Os*CGT | 22 | 16.8 ± 0.1 | 90 ± 1 | 92 ± 1 |
|  |  |  | *Gm*SuSy | 18 | 14.2 ± 0.1 | 93 ± 1 | 91 ± 1 |
|  | 144 | 15.9 ± 0.2 | *Os*CGT | 26 | 17.6 ± 0.2 | 89 ± 1 | 86 ± 2 |
|  |  |  | *Gm*SuSy | 22 | 15 ± 0.2 | 88 ± 1 | 89 ± 2 |
|  | 162 | 16.7 ± 0.1 | *Os*CGT | 29 | 18.5 ± 0.5 | 83 ± 1 | 76 ± 2 |
|  |  |  | *Gm*SuSy | 24 | 16.4 ± 0.1 | 83 ± 2 | 82 ± 2 |
|  | 200 | 18.8 ± 0.3 | *Os*CGT | 36 | 19.7 ± 0.1 | 78 ± 2 | 70 ± 2 |
|  |  |  | *Gm*SuSy | 30 | 18.2 ± 0.2 | 79 ± 3 | 76 ± 2 |
|  | 240 | **19.8 ± 0.5**^[e]^ | *Os*CGT | 43 | 20.5 ± 0.5 | 74 ± 4 | 65 ± 4 |
|  |  |  | *Gm*SuSy | 36 | 18.9 ± 0.3 | 74 ± 2 | 71 ± 2 |

^[a]^ The loading ratio Z-*Os*CGT/Z-*Gm*SuSy is shown.

^[b]^ The total enzyme loading of co-immobilized enzyme.

^[c]^ The observable activity for the co-immobilized enzyme preparation is shown. Reaction conditions: 1.0 mM phloretin, 500 mM sucrose, 0.5 mM UDP, 50 mM KCl, 13 mM MgCl_2_, 1.3 mg/ml BSA, 20% DMSO, 30°C, 50 mM HEPES buffer (pH 7.5); 30°C; analysis by HPLC for nothofagin formed.

^[d]^ The observable activity for the individual immobilized enzyme preparation is shown. For Z-*Os*CGT, reaction conditions: 1.0 mM phloretin, 2.0 mM UDP-glucose, 50 mM KCl, 13 mM MgCl_2_, 4% DMSO, 30°C, 50 mM HEPES buffer (pH 7.5); For Z-*Gm*SuSy, reaction conditions: 500 mM sucrose, 2.0 mM UDP, 50 mM KCl, 13 mM MgCl_2_; 50 mM BisTris buffer (pH 6.5).

^[e]^Unless mentioned, this was the preparation of co-immobilized enzymes used in the continuous flow experiments for nothofagin production. It is therefore highlighted in bold.

Mean values of three independent determinations and the corresponding standard errors are shown.

**SUPPORTING FIGURES**

**
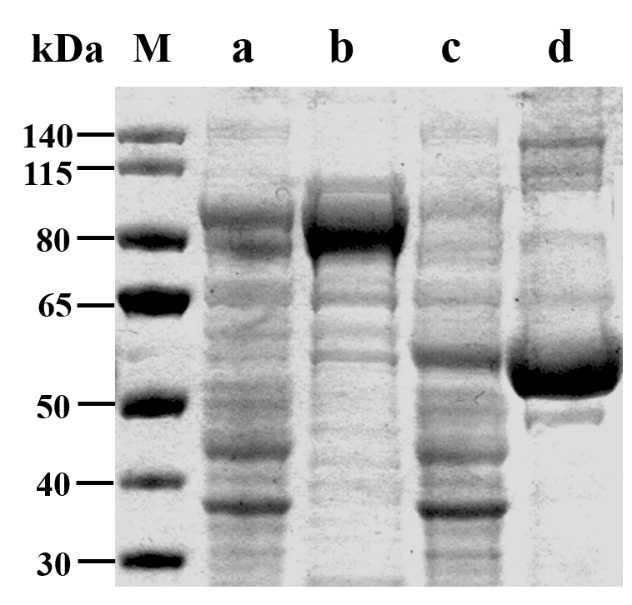
**

**Figure S1.** SDS polyacrylamide gel showing the preparations of Z-*Gm*SuSy and Z-*Os*CGT used. M: PageRuler^TM^ Prestained Protein Ladder (Thermo Scientific); a: *E. coli* lysate of Z-*Gm*SuSy; b: purified Z-*Gm*SuSy; c: *E. coli* lysate of Z-*Os*CGT; d: purified Z-*Os*CGT. Enzymes were purified by cation exchange chromatography (Liu et al. 2021)^[a]^ and their apparent molecular masses were in accordance with expectations for the full-length protein containing the Z_basic2_ module (Z-*Os*CGT: 57849 Da; Z-*Gm*SuSy: 100661 Da, enzyme subunit).

^[a]^ Liu, H., Tegl, G., Nidetzky B. *Adv. Synth. Catal.* **2021**, *363*, 1 –14.


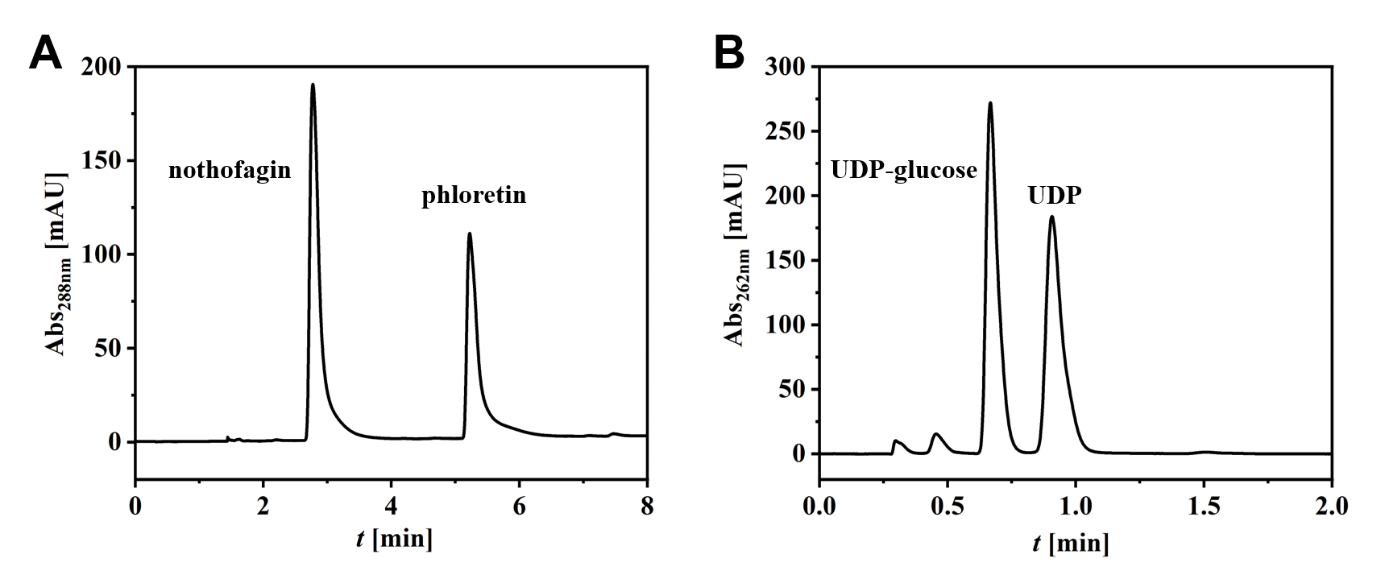


**Figure S2.** Representative absorbance traces of the HPLC analyses used. A: Reversed phase C-18 HPLC with detection at 288 nm for analysis of phloretin (1 mM) and nothofagin (1 mM). B: Ion-pairing reversed phase C-18 HPLC with detection at 262 nm for analysis of UDP (2 mM) and UDP-glucose (2 mM). The sample volume was 10 µl.

Methods used: A Shimadzu model UFLC HPLC system equipped with a Kinetex^®^ 5 µm EVO C18 LC column (100 Å, 150 × 4.6 mm; Phenomenex, Aschaffenburg, Germany) was used. The column was equilibrated at 25°C in 20 mM potassium phosphate buffer (pH 5.9). Elution was with acetonitrile at 1 ml/min. The sample volume was 10 µl. Samples were diluted to a concentration of about 0.5 – 1.0 mM (phloretin, nothofagin) and 0.5 – 1.5 mM (UDP, UDP-glucose). To analyze nothofagin and phloretin, an ascending acetonitrile gradient from 25% to 60% was used over 7 min. To analyze UDP-glucose and UDP, the ion-paring reagent tetrabutylammonium bromide (40 mM) was added to the phosphate buffer. Isocratic elution at 12.5% acetonitrile was used.

**
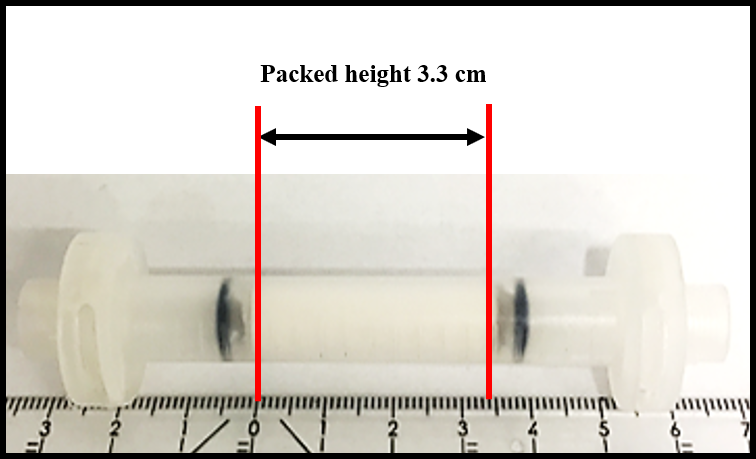
**

**Figure S3:** The packed-bed column used for continuous reaction. Total volume, 1.0 cm^3^; diameter, 0.62 cm; packed height, 3.3 cm.


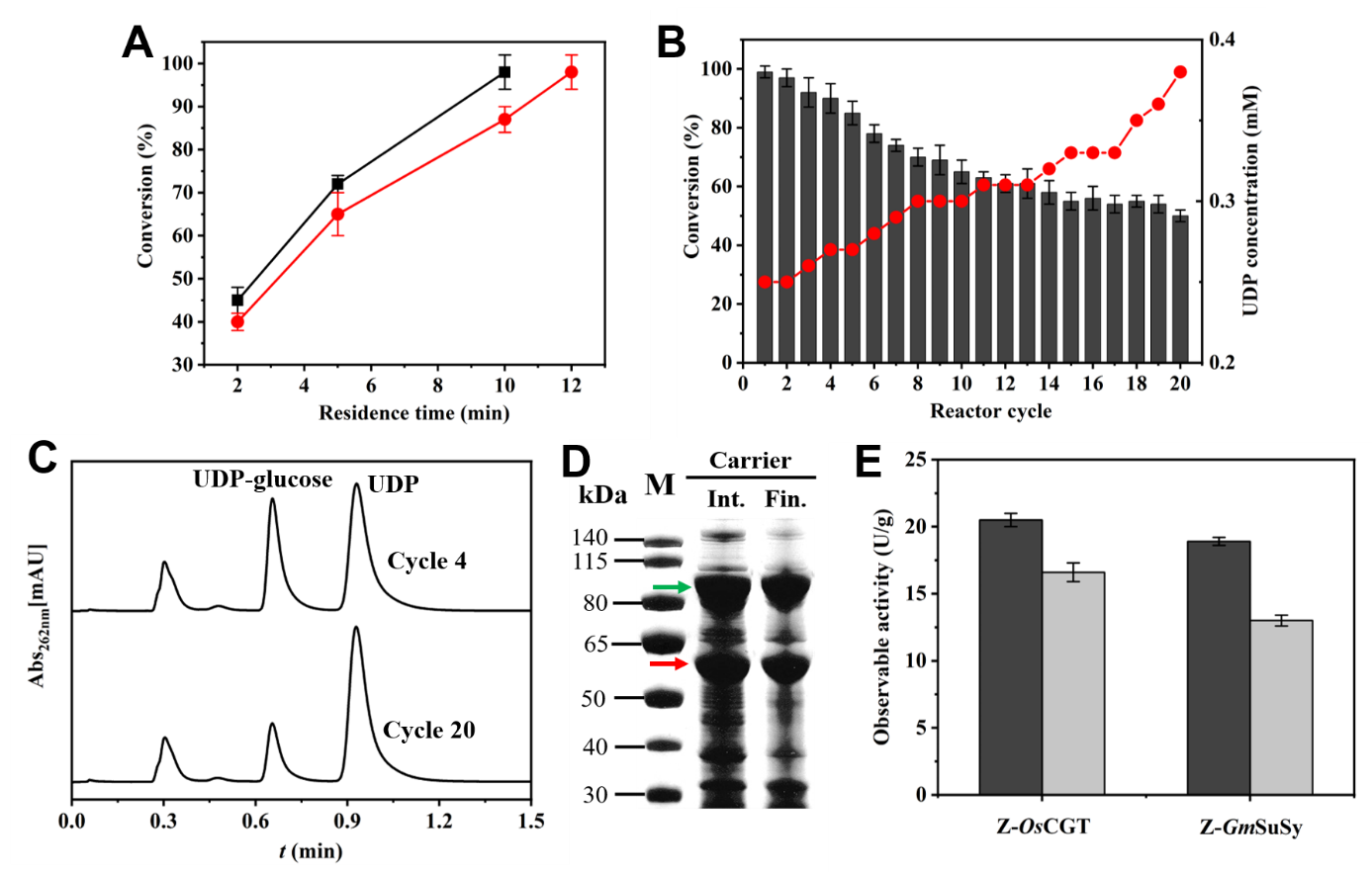


**Figure S4.** Effect of DMSO co-solvent on co-immobilized enzyme activity and stability for flow synthesis of nothofagin. Panel **A** shows reaction at different τ_res_ using 10 mM phloretin solubilized with 2-hydoxypropyl-β-cyclodextrin (black squares) or 20% (by volume) DMSO (red circles). The other conditions were: 500 mM sucrose, 0.5 mM UDP, 1.3 mg/ml BSA, 50 mM KCl, 13 mM MgCl_2_, 50 mM HEPES buffer (pH 7.5); 30°C. The enzyme preparation with an observable activity of 19.8 U/g was used (Table S3). Panel **B** shows continuous reaction (τ_res_ = 12 min) under the stated conditions of panel A using 20% DMSO. The bars show the phloretin conversion into nothofagin and the red circles show the UDP concentration. Panel **C** shows HPLC absorbance traces of samples analyzed for UDP and UDP-glucose content after a certain number of reaction cycles. Note: the sum of UDP-glucose and UDP concentrations equals the added UDP concentration of 0.5 mM. Panel **D** shows a SDS polyacrylamide gel of the protein bound to the ReliSorb SP400 carrier at reaction start (Int.) and at reaction end (Fin., cycle 20, 240 min). Lane M shows molecular mass markers. The green arrow indicates Z-*Gm*SuSy, the red arrow Z-*Os*CGT. Panel **E** shows the individual enzyme activities on the ReliSorb carrier at reaction start (dark gray bars) and after cycle 20 (light gray bars).

The values shown are means of three independent determinations with their corresponding standard errors.

**
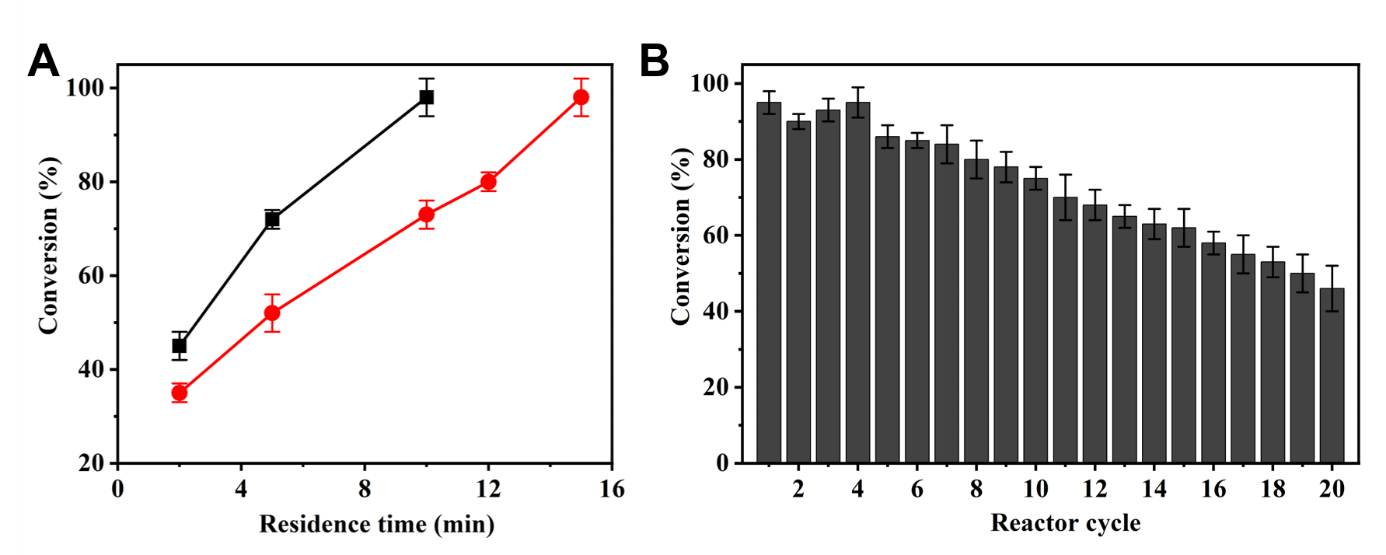
**

**Figure S5.** Effect of added cations on co-immobilized enzyme activity and stability for flow synthesis of nothofagin. Panel **A** shows reactions in the presence (black squares) and absence (red circles) of 50 mM KCl and 13 mM MgCl_2_. The other conditions were: 10 mM phloretin (solubilized with 2-hydroxypropyl-β-cyclodextrin), 500 mM sucrose, 0.5 mM UDP, 1.3 mg/ml BSA, 50 mM HEPES buffer (pH 7.5); 30°C. The enzyme preparation with an observable activity of 19.8 U/g was used (Table S3). Panel **B** shows continuous reaction (τ_res_ = 15 min) under the stated conditions of panel A in the absence of cations. The figure can be compared with Figure 3c of the main text. The half life of the catalyst (here: ~280 min) was drastically decreased in the absence of added KCl and MgCl_2_.


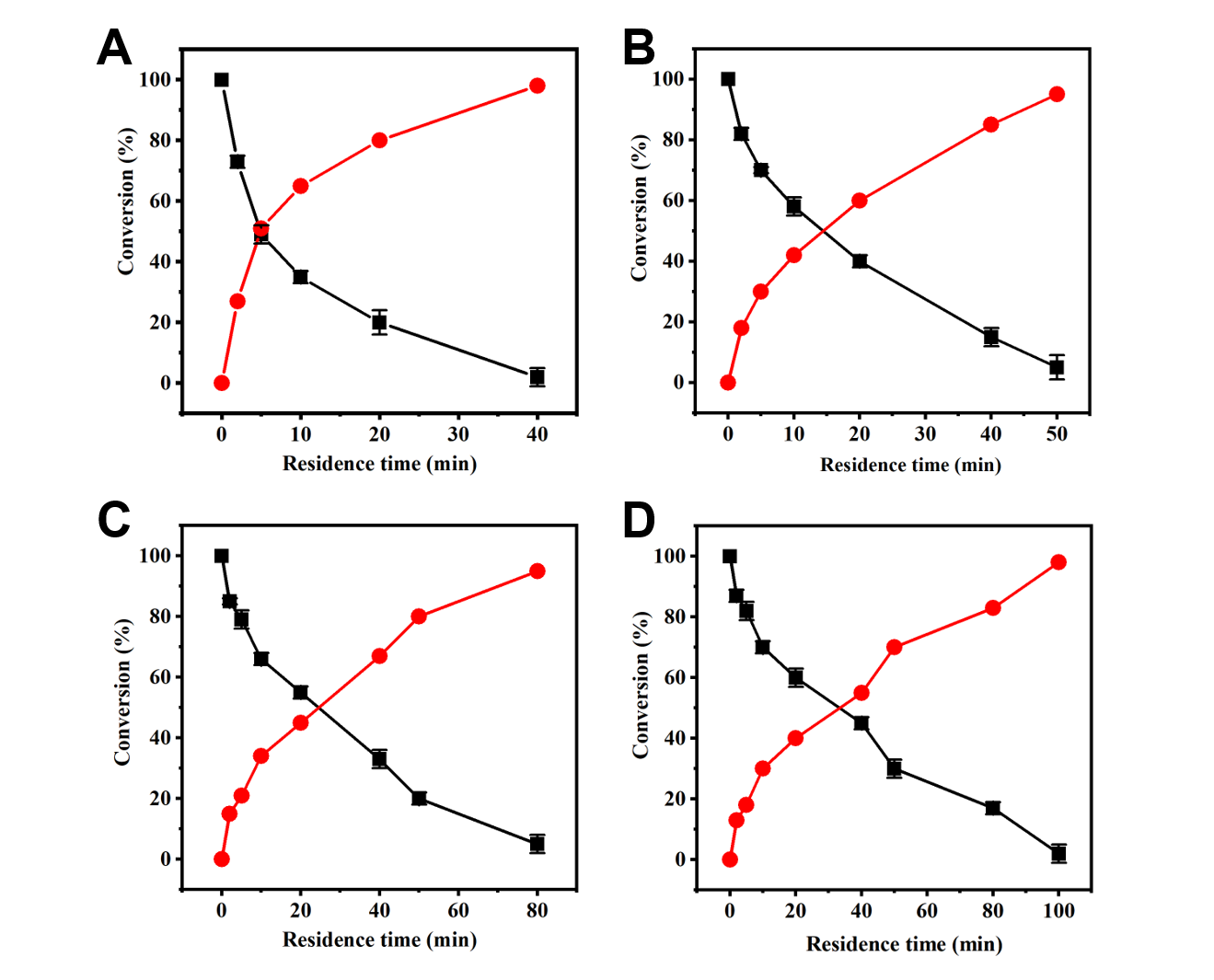


**Figure S6.** Flow synthesis of nothofagin (circles, red) from phloretin (squares, black) solubilized as inclusion complex with 2-hydroxypropyl-β-cyclodextrin (**A**, 20 mM; **B**, 30 mM; **C**, 40 mM, **D**, 50 mM) using co-immobilized Z-*Os*CGT and Z-*Gm*SuSy. Reaction conditions: 500 mM sucrose, 0.5 mM UDP, 50 mM KCl, 13 mM MgCl_2_, 1.3 mg/ml BSA, 50 mM HEPES buffer (pH 7.5); 30°C. The enzyme preparation with an observable activity of 19.8 U/g was used (Table S3).
